# A novel deletion in the *BLOC1S6* Gene Associated with Hermansky-Pudlak syndrome type 9 (HPS-9)

**DOI:** 10.1186/s12864-024-10478-w

**Published:** 2024-08-27

**Authors:** Seyyed Mohammad Kahani, Ali Rabbizadeh Saray, Mir Salar Kahaei, Ali Dehghani, Pouria Mohammadi, Masoud Garshasbi

**Affiliations:** 1 Department of Medical Genetics, Faculty of Medical Sciences, Tarbiat Modares University, Tehran, Iran; 2 Department of Medical Genetics and Molecular Medicine, Faculty of Medicine, Mashhad University of Medical Sciences, Mashhad, Iran; 3 PardisGene CO, Tehran, Iran

**Keywords:** Albinism, Hermansky-pudlock syndrome type 9, Whole exome sequencing, Primer-walking, GAP-PCR

## Abstract

**Background:**

Hermansky-Pudlak Syndrome (HPS), a rare autosomal recessive disorder, is characterized by oculocutaneous albinism, bleeding diathesis, and sometimes severe lung problems and inflammatory bowel disease. Symptoms include skin and hair pigmentation variations, along with visual impairments. Variants in eleven genes encoding protein complexes essential for membrane trafficking and intracellular endosomal transport pathways underlie various recognized HPS subtypes. This study focuses on HPS-9, a subtype of Hermansky-Pudlak Syndrome caused by a variant in the *BLOC1S6* gene, which is a subunit of the BLOC1 complex. In this study, a novel Copy Number Variation (CNV) in the aforementioned gene in an Iranian family is reported. The study aims to better understand the etiology of HPS-9 symptoms by identifying and confirming the variant and determining whether the gene is expressed despite the deletion. There have only been five reports of this syndrome in the literature thus far. Our novel CNV represents a significant contribution to understanding the genetic basis of HPS-9.

**Results:**

This study investigates a male patient presenting with albinism. Whole Exome Sequencing (WES) identified a homozygous deletion of approximately 350 bp using CNV analysis. The deletion affects the intronic region of the *BLOC1S6* gene, causing uncertainties in defining the exact boundaries due to WES limitations. Primer walking and GAP-PCR techniques were used to define the deletion boundaries. Subsequent assessments of this variant across other family members helped identify homozygous affected members and heterozygous carriers. The absence of *BLOC1S6* expression in the affected individual was confirmed through Real-time PCR experiments. These findings underscore the importance of understanding the implications for the patient’s healthcare and potential therapeutic approaches.

**Conclusion:**

This study introduces a case of Hermansky-Pudlak Syndrome Type 9 (HPS-9) caused by a homozygous deletion in the *BLOC1S6* gene. We identified an approximately 7-kb deletion encompassing exon 1 and the intronic region of the gene. The absence of *BLOC1S6* expression, confirmed via Real-time PCR, highlights the importance of studying the pathogenicity of the deletion and its impact on the patient’s health. Our findings contribute to the sparse knowledge on HPS-9 and underscore the need for further exploration into the genetic causes of this rare disorder.

## Background

Albinism refers to a group of conditions that result from defects in the production of melanin and the biogenesis and transport of melanosomes. Melanin is primarily responsible for the pigmentation of the skin, hair, and eyes, and it plays a crucial role in protection against the harmful effects of sunlight. Consequently, a decrease in the pigmentation of these organs is the primary indication of albinism [1, 2]. Albinism is classified into two distinct types: oculocutaneous albinism (OCA), which affects the eyes, skin, and hair, and ocular albinism (OA), which only affects the eyes. OCA is further subdivided into two main categories: non-syndromic, which displays alterations in the pigmentation of the aforementioned organs, and syndromic forms such as Hermansky-Pudlak Syndrome and Chediak-Higashi Syndrome (CHS). Syndromic forms exhibit additional systemic complications in addition to the classical OCA phenotype. The manifestations of non-syndromic and syndromic forms depend on the mutated gene [1, 3]. If the gene is only involved in the production and function of melanosomes, non-syndromic forms of OCA will arise. Conversely, variants in the genes involved in the cargo trafficking of lysosome-related organelles (LROs) will result in syndromic forms [4].

HPS is classified as part of the albinism group of disorders because of its classical OCA phenotype, which is caused by defects in the biogenesis of melanosomes. Furthermore, the impaired biogenesis of platelet-dense granules is the cause of the excessive bleeding and bruising in HPS. In some subtypes of HPS, defects in the biogenesis of lamellar bodies can lead to progressive lung fibrosis due to reduced surfactant levels [5].

HPS is a genetically heterogeneous, rare, autosomal recessive disorder that can be caused by inactivating variants in 11 genes that encode the subunits of the four obligatory multi-subunit protein complexes [6]. These proteins include membrane/intracellular endosomal trafficking complexes, known as the Biogenesis of Lysosome Organelle Complex (BLOC-1, BLOC-2, BLOC-3), and the adaptor protein (AP-3) [4, 5]. Impairment in the function of any of the polypeptide constituents of the mentioned proteins can cause a different subtype of HPS. Therefore, 11 subtypes of HPS have been identified to date. BLOC1 consists of eight subunits referred to as BLOC1S1-8 [5]. HPS-9, a subtype of Hermansky-Pudlak Syndrome, is caused by a variant in the *BLOC1S6* gene, also known as *PALLID*. This gene is approximately 22 kilobases in length and is located on the ‘q’ arm of chromosome 15. This gene produces a transcript, denoted as NM_012388.4, which is 3,778 base pairs long. This transcript is segmented into five distinct exons [7].

In human beings, the *PALLID* gene transcripts, have low tissue specificity and is ubiquitously expressed across various tissues, with the most prominent expression noted in skeletal muscle, according to data from the Protein Atlas database and the FANTOM5 dataset [8].

The *BLOC1S6* gene is reported to produce multiple alternatively spliced transcripts that encode distinct protein isoforms. Three protein-coding transcripts are identified for the *BLOC1S6* gene. Transcript NM_001311255.1 encodes isoform 1, consisting of 177 amino acids. The canonical transcript NM_012388.4 generates isoform 2 with 172 amino acids, differing in its 5’ UTR and coding region, and initiates translation from an alternative start codon compared to NM_001311255.1. Transcript NM_001311256.1 is notable for a deletion in the central coding region that causes a frameshift and premature stop codon, resulting in isoform 3, which features a distinct and shorter C-terminus relative to isoform 1 [9].

The protein product, pallidin, a homodimer with a molecular mass of approximately 19,744 Da and consisting of 172 amino acids, is a crucial component of the Biogenesis of Lysosome-related Organelles Complex-1 (BLOC-1). This complex is implicated in the regulation of endosomal membrane fusion, interacting with the t-SNARE syntaxin 13. This interaction is vital for the fusion process during melanosome maturation, a critical pathway elucidated in cases of Hermansky-Pudlak syndrome type 9, which is a rare syndromic form of albinism marked by a severe defect in platelet-dense bodies [10, 11].This functional characterization highlights the complex regulatory mechanisms that orchestrate cellular compartmentalization and the systemic implications of their dysregulation.

According to the literature, to date, only five cases of this syndrome have been reported. These cases are characterized by single nucleotide variants (SNV) variations. Notably, two of these cases were due to compound heterozygous variants. No copy number variations (CNV) have been reported for this gene so far. According to the OMIM database, this syndrome presents with symptoms such as ocular albinism, nystagmus, cutaneous albinism, thrombocytopenia, leukopenia, and recurrent cutaneous infection [12].

In this study, a pedigree with members exhibiting symptoms of albinism from East Azerbaijan Province, Iran, was investigated. Using whole-exome sequencing (WES), it was found that the patients have a large deletion in the *BLOC1S6* gene. Subsequently, the boundaries of this deletion were determined proximally using the primer walking method that finally confirmed by G-PCR. Additionally, we performed a comparative analysis of *BLOC1S6* expression levels between the proband and a healthy control individual. with implications for understanding and treating albinism and related disorders, this study presents an analysis of the *PALLID* gene and its involvement in HPS development.

## Methodology and materials

Ethical considerations were given priority throughout this study. All participants were adequately informed about the research objectives and their consent was obtained. The study was approved by the medical ethics committee at Tarbiat Modares University, in Tehran, Iran (IR.MODARES.REC.1400.307).

### WES and bioinformatics analysis

Genomic DNA was extracted from participants’ blood samples using the standard salting-out procedure. Subsequently, DNA samples were enriched using the Twist Human Core Exome Plus Kit. The generated library was then sequenced on the NovaSeq 6000 platform, resulting in a mean coverage of 100X and generating 8 gigabytes of data.

Quality control checks on read length and depth, based on GC content (50%) and Phred score (Phred = 20), were crucial in identifying potential errors introduced during library preparation and sequencing stages. These checks were performed using the FastP tool [13], generating an HTML-formatted QC report.

The FASTQC software was used to assess the quantity and quality of the sequencing reads. Consequently, the sequencing data for mapping was aligned to the reference genome (GRCh38/hg38) using the Burrows-Wheeler aligner (BWA-MEM), which generated a Sequence Alignment Map (SAM) file. We used Samtools (Li et al., 2009) to convert the SAM file into a BAM file [14]. Following this, the BAM file and its index file were used to visualize read depth and variant localization through the Integrated Genome Viewer (IGV) software [15, 16].

Calling germline single nucleotide polymorphism (SNP) and insertion/deletion (Indel) variants was performed using the Genome Analysis Toolkit HaplotypeCaller (GATK HC). The output of this step was a variant call format (VCF) file, which contained both SNP and Indel variants [17].

**Fig. 1:**
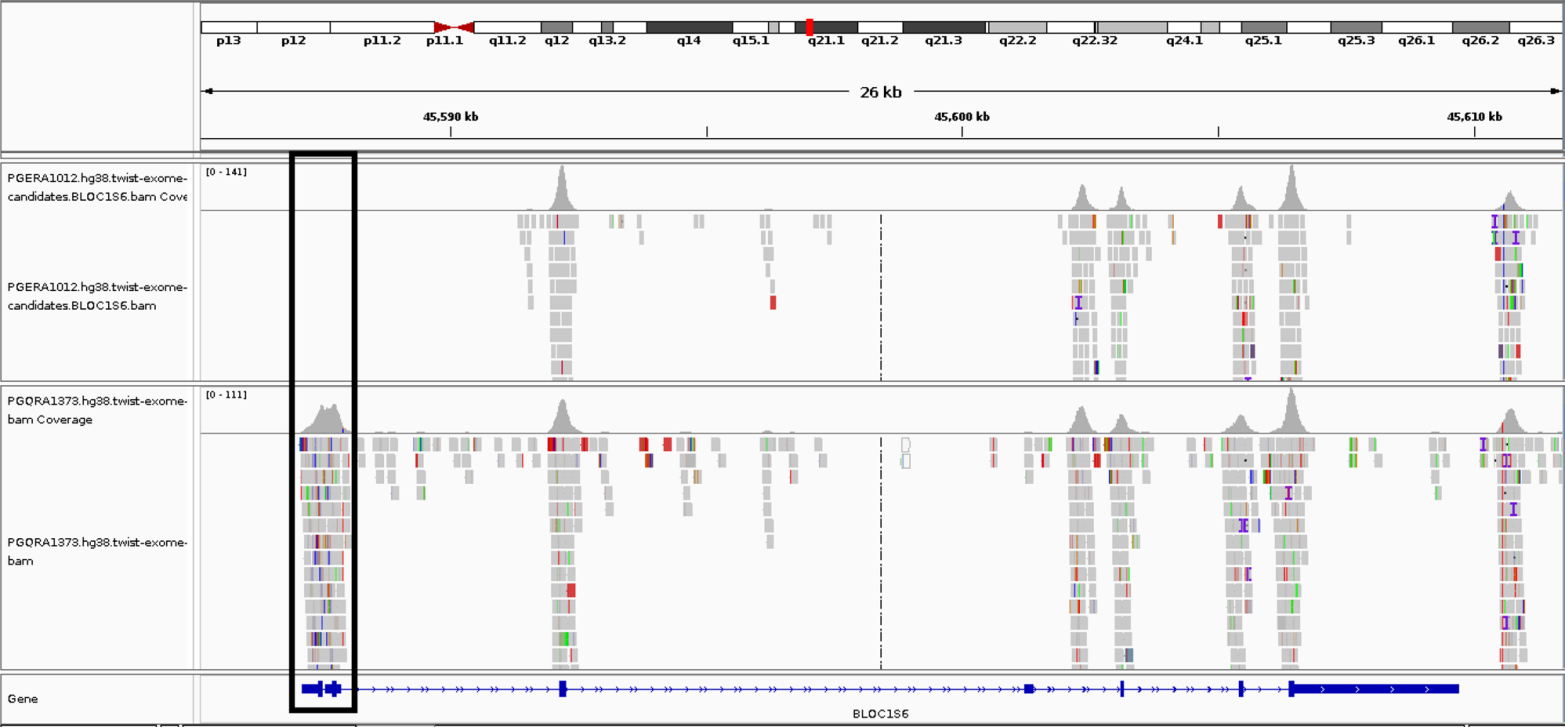
Comparison of the BAM file of the proband with the BAM file of a healthy control individual. The upper row corresponds to the proband and the lower row corresponds to the control. The deletion is evident for the proband within the region specified. The figure was generated from the IGV software

We also used the XHMM tool [18] and the ExomeDepth [19] method for detecting copy number variations (CNVs). The results of these CNV’s were then compared with those from control samples to conduct a comprehensive analysis of the genomic variants present in our sample. Furthermore, our research included a comparison of the BAM file of the individual under study using the IGV software, along with comparisons made with control samples. This analysis revealed a notable deletion of exon1 within the BAM file of the patient (Fig. 1).

The Variant Effect Predictor (VEP) played a pivotal role in annotating variants and providing functional insights.

Filtering common variants was performed using databases such as the Genome Aggregation Database (gnomAD v3), the Exome Aggregation Consortium (ExAC v1), and Iranome. Furthermore, we also excluded benign variants, following the guidelines provided by the American College of Medical Genetics (ACMG). Variants that were already present in clinical databases, such as the Human Gene Mutation Database (HGMD) professional and ClinVar, were considered.

Subsequently, variants in genes associated with the patient’s disorder were considered using Human Phenotype Ontology (HPO) terms.

Furthermore, the Online Mendelian Inheritance in Man (OMIM) database was used to identify diseases linked to the genetic variants found in the patient.

### Primer walking and Gap-PCR

For detailed genetic analysis surrounding the *BLOC1S6* gene, primer walking and GAP-PCR techniques were employed. Primer walking is a method that allows for the sequencing of long DNA templates, addressing the limitation of the Sanger chain termination method, which can only sequence a few hundred bases at a time. The process involves an iterative approach, starting with an initial round of sequencing from a known sequence at one end of the DNA template. Each subsequent round is initiated with a new primer, which is based on the end of the sequence obtained from the previous round [18].

**Fig. 2:**
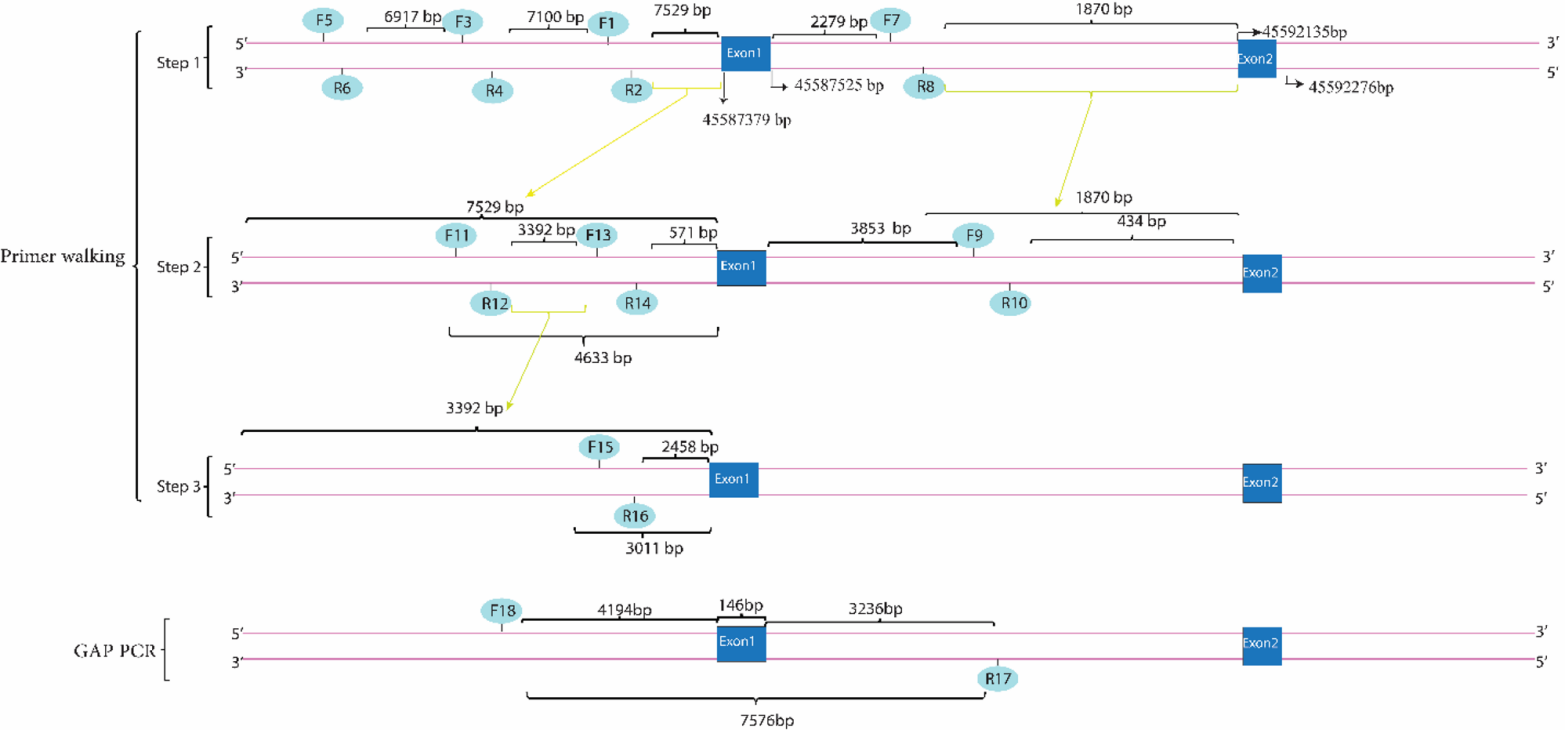
Illustrates the primer walking process followed by GAP-PCR to identify the boundaries of a genomic deletion. The primer pairs (1–2, 3–4, 5–6) were designed to target regions upstream of the first exon of the *BLOC1S6* gene, successfully amplifying their target regions. The breakpoint was determined using primer pair 11–12, which amplified the expected PCR product. Primer pairs 13–14 and 15–16 were designed to further delineate the deletion boundaries and were found to be within the deleted region. Primer pairs 7 and 8 were then designed for the downstream deletion boundary, and primer pair 9–10 was used to approach exon 2. The data from primer walking led to the hypothesis that primers 10 and 11 could be used for the GAP-PCR method. The resulting PCR product was subjected to Sanger sequencing, and based on the sequence, another reverse primer [15] was designed. The GAP-PCR method was applied using primers 11 and 17, which produced an 800-base-pair PCR product confirmed by gel electrophoresis. We performed bidirectional Sanger sequencing on the PCR product until the results were clear and free of noise. To achieve a 450-base-pair PCR product, We designed a forward primer 18, which was placed 439 bases downstream of primer 11. We then used this forward primer 18, along with reverse primer 17, through the GAP-PCR method. The corresponding reference genomic sequence for this region was found to be approximately 7 kb in length, and the GAP-PCR based test confirmed the presence of the deletion  (Please note that while the numerical scales indicated in this image, such as base pair (bp) measurements, are accurate and reflect true sizes of DNA fragments and genomic features, the physical dimensions of the image itself are not to scale)

Multiple primers were designed to target specific intergenic and intronic regions both upstream and downstream of the *BLOC1S6* gene as outlined in our approach (Fig. 2). These regions were systematically selected to ascertain the presence of any genomic deletions that could influence gene function.

In our case, primers were designed to match both sides of the target DNA sequences, which we knew, based on WES data, had not been deleted. This approach was continued until a suitable region for generating a PCR product spanning the deleted region was found. Finally, we used primers on both sides of the deletion area, using the GAP-PCR method.

The primers were designed using the Primer Blast tool [20], and their specificity was verified through this platform. The potential presence of single nucleotide polymorphisms (SNPs) at the 3’-end of both forward and reverse primers at each step of primer walking was verified using the Genetools website [21] and the SNP 151 in-silico PCR tool of the UCSC genome browser [22]. Given significant distance length of the region between the *BLOC1S6* gene and the upstream genes, and the uncertainty surrounding the precise distance at which the PCR product would be obtained, a stepwise approach for primer design was developed (Table 1).

**Table 1:** Sequence and positioning of the different primers designed

Primer Name	Primer Sequence(5’→3’)	Location
Forward Primer (1)	TTACCCACCTTCTCCCTTTAGGT	Upstream
Reverse Primer (2)	TACAGCAGTGACACAGATTAAATGG	
Forward Primer (3)	TGTGAAAACCCCAAAGTGGAAGA	Upstream
Reverse Primer (4)	GAATATCGGAGGGCTTGGCA	
Forward Primer (5)	CACAACCAAGTGGGAAACATAGC	Upstream
Reverse Primer (6)	ACTGATGTTCTGCCTGTTCTGA	
Forward Primer (7)	TTCTTAACCATTGGAAGCAGAGGG	Downstream
Reverse Primer (8)	TGCAAGGCAACATTCAAATAGGTA	
Forward Primer (9)	CAGTGACCGTTACCCACGTT	Downstream
Reverse Primer (10)	TCAGGGCAGAGGACTTATAGTTT	
Forward Primer (11)	TGTGTGACGCAGACTTTACAGAT	Upstream
Reverse Primer (12)	CACAATCCCACTACTCCTTTCAT	
Forward Primer (13)	ATAAAGGCAATGGGGAACCAGT	Upstream
Reverse Primer (14)	TGCAGCAGCTACTGAACAAATTC	
Forward primer (15)	GGAGACTTCAGGGTCACAGG	Upstream
Reverse Primer (16)	AAACCTAGGTCTTGGGCCAG	
Reverse Primer (17)	GATTGAGGAAAACAACATGCTATC	Downstream
Forward Primer (18)	GAGCCTTGGAAGAGAAGTAGGA	Upstream

We expanded the target region of GAP-PCR to its maximum extent, as feasible. A comprehensive segregation analysis was performed within the pedigree under study, with the aim of identifying family members who carry the variant but do not exhibit the associated phenotype.

### Real time PCR

Subsequently, we aimed to ascertain whether the *BLOC1S6* gene still shows expression in the blood of the homozygous index patient, despite the deletion in exon 1. This study aimed to identify any alternative transcripts for the gene in the absence of exon 1. Real-time PCR was applied to analyze the transcripts of *BLOC1S6* starting from the second exon. The primers were designed using the IDT oligo web-based tool, with a focus on the two main transcripts of this gene and the deletion in exon 1. The forward primer was designed for exon 2, and the reverse primer spanned the junction between exon 3 and exon 4. The primer sequences are as outlined below: Forward Primer: 5’- GAGCAACTGGCAGAAGGATTG − 3’ (exon 2) and Reverse Primer: 5’- GCCTCAGCAAACAAAGCATTAATATC − 3’ (exon 3 and 4). The PCR reactions were performed with an initial denaturation step at 95 °C for 10 min, followed by 45 cycles of denaturation at 95 °C for 20 s, annealing at 60 °C for 20 s, and extension at 72 °C for 40 s.

Real-time PCR tests were performed in duplicate for each sample. The reaction mix for each sample consisted of 3 µl of cDNA, 10 µl of master mix, 1 µl each of forward and reverse primers, and 5 µl of distilled water, resulting in a total reaction volume of 20 µL. Furthermore, Glyceraldehyde-3-Phosphate Dehydrogenase (*GAPDH*) was used as the internal control. The primer sequences targeting *GAPDH* are given below:

Forward Primer: 5’- GTCTCCTCTGACTTCAACAGCG − 3’ (exon 8) and Reverse Primer: 5’- ACCACCCTGTTGCTGTAGCCAA − 3’ (exon 9).

## Results

### Clinical findings

The proband is a thirty-two-year-old man originally from Khodafarin, a locality within East Azerbaijan province in Iran. The patient’s lineage is noteworthy for the practice of distant relative consanguinity, a factor that significantly contributes to the genetic context of the study.

The patient’s father, who passed away at the age of 68, was reported to be free from hereditary diseases and experienced a natural death, suggesting no direct genetic predispositions from this lineage. Conversely, the mother, currently aged 58, is also from a background of distant consanguinity, which could imply a potential for genetic conditions inherited maternally. The proband’s spouse, a 34-year-old woman, shares a similar background of distant relative consanguinity, adding another layer of genetic complexity to the family tree.

Moreover, the proband’s immediate family and extended relatives present a spectrum of clinical conditions that merit attention. Notably, his brother, niece, and the grandson of his grandfather-in-law exhibit the albinism phenotype. The presence of albinism within the family underscores the presence of genetic variability that could be pivotal for the study. Additionally, the possibility of other carriers or individuals affected by similar or different genetic conditions within the local area has been highlighted, suggesting a broader investigation into the genetic landscape of this community.

The population size of Khodafarin, while not specified, is indicative of a small community where genetic traits and conditions could be more easily traced and studied due to the closer kinship ties and reduced genetic variability compared to larger populations. This environmental and social setting provides a unique opportunity to explore the genetic interplay between consanguinity, inherited conditions, and their manifestation within a specific demographic.

**Fig. 3:**
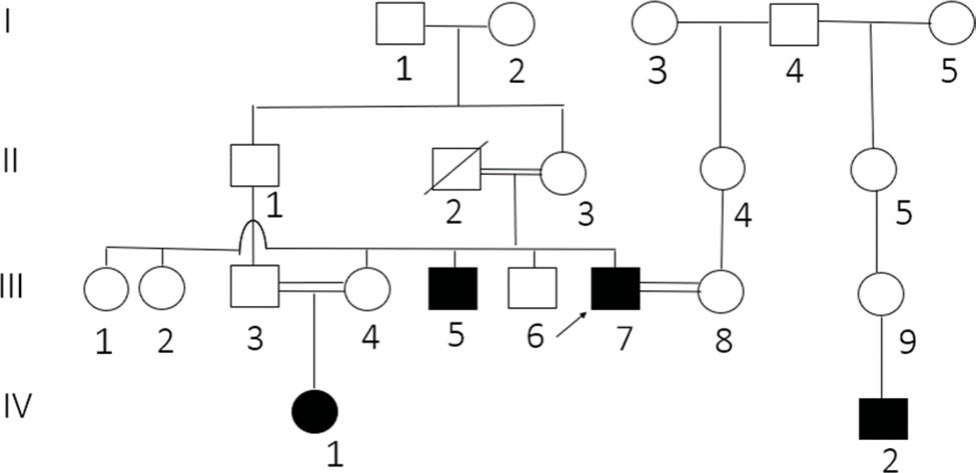
The index case, highlighted by an arrow, was a 32-year-old male with parents who were distant relatives by consanguinity. His 68-year-old father, who remained healthy until his natural death, and his 58-year-old mother are notable family members. The proband married a 34-year-old woman, who also had a family history of distant consanguinity. Originating from a small village in Iran, this case is further intriguing due to the presence of the albinism phenotype not only in his brother and niece but also in the grandson of his grandfather-in-law. In addition to the index patient and his immediate family, it is noted that another distant relative appears to be unrelated to the proband. However, the presence of consanguinity issues within the family suggests that there may be other carriers and affected individuals residing in the village. This observation underscores the potential for a broader impact of genetic disorders within the community, highlighting the importance of further investigation to identify and understand the extent of the genetic predisposition among the population

The proband has displayed symptoms of albinism, such as light pink skin, white-blonde hair, and brittle skin. His brother exhibited symptoms of albinism besides frequent nosebleeds. In contrast, the phenotype of his brother, his parents and sisters were normal. Interestingly, the proband’s niece and his wife’s paternal half-cousin also showed evidence of albinism, suggesting that the condition may not just affect members of the immediate family. Unfortunately, they could not participate in this study because of access issues. Regretfully, the father had already died before the research began. Consanguineous marriage between the proband’s parents may have contributed to his family’s genetic susceptibility to albinism (Fig. 3).

### WES results

Our investigation into single nucleotide variants (SNVs) and insertion/deletion variants (indels) did not uncover any pathogenic or likely pathogenic variants that could be linked to the albinism phenotype. We conducted a more thorough examination, incorporating the analysis of CNVs. This allowed us to accurately detect a deletion using the eXome-Hidden Markov Model (XHMM), providing a high level of confidence. The detection was further validated by the ExomeDepth method, which also offered a high level of certainty. Both methods detected a homozygous deletion of approximately 350 bp in the region DEL: chr15:?-45587443_45587796-? compared to the control samples. However, the limitation of whole exome sequencing in covering intronic regions prevented us from detecting the exact region.

Further validation of the deletion was conducted by performing a comparative analysis of the patient’s BAM files and those of a healthy control individual. Both samples were processed using the Twist Human Core Exome Plus kit. The analysis revealed a notable deletion in exon 1 of the patient’s sample, which was not observed in the sample from the healthy individual. The deletion was then evaluated in the Franklin database, which assigned it a pathogenicity score of 0.9. This score indicates a high probability of the deletion being pathogenic.

In summary, the WES analysis did not identify any pathogenic or likely pathogenic variants related to albinism in the SNVs. However, a homozygous likely Pathogenic deletion was identified on the long arm of chromosome 15 in the CNV analysis.

This deletion also affects the intronic region, leading to uncertainties in determining the exact boundaries and size of the deleted region. These uncertainties result from the inherent limitations of the WES method in capturing intronic regions, highlighting the necessity of an additional confirmatory test.

### Primer walking and GAP-PCR

**Fig. 4:**
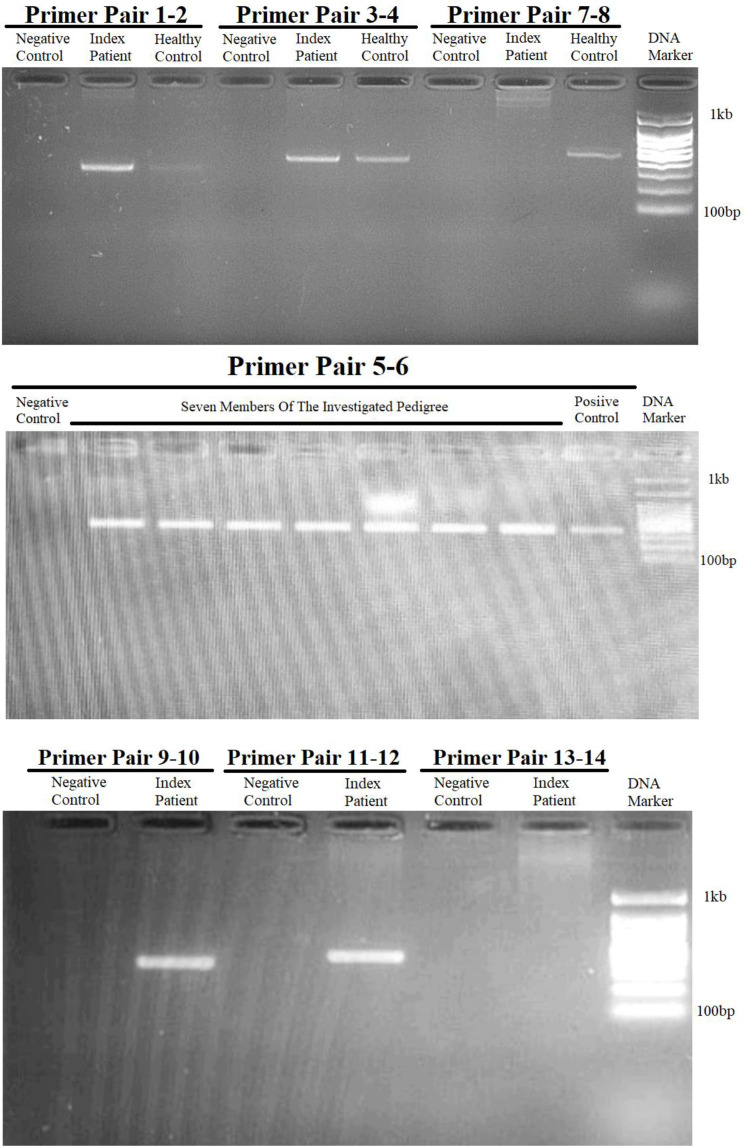
Results of the gel electrophoresis after performing PCR using primer pairs targeting the flanking regions of exon 1 of the *BLOC1S6* gene. It is evident, the results of PCR using all primer pairs targeting the upstream region of exon 1 of the *BLOC1S6* gene, including primer pairs 1–2, 3–4, 5–6, and 11–12 are positive for the index patient (proband) and the healthy control. this indicates that the targeted loci are not deleted. However, the results for primer pair 13–14, that this pair targets a locus within the deleted region. On the other hand, the result of PCR using primer pair 9–10 targeting the downstream region of exon 1 of the *BLOC1S6* is positive for the index patient and the healthy control, whereas the result of PCR using primer pair 7–8 used in the first try to target the downstream side was negative for the index patient, which indicates that this locus is located in the deletion region

Utilizing primer walking and GAP-PCR techniques, multiple primer pairs to amplify specific regions around the *BLOC1S6* gene were designed, as depicted in Fig. 2. In the first step, primer pairs 1to 6 targeted the upstream intergenic regions adjacent to *BLOC1S6* and successfully amplified these regions, confirming their integrity and absence of deletions (Fig. 4). However, primer pairs 7 and 8, positioned 2279 base pairs from exon 1, failed to yield expected results, indicating their location within a possible deletion zone (Figs. 2 and 4).

Next step involved primer pairs 11–12 and 13–14, designed to further explore the intronic and intergenic spaces. Primer pair 11–12, located 2896 base pairs downstream primer pair 1–2 and 4633 base pairs upstream exon 1, successfully amplified the target, suggesting that it lies outside the deleted region. In contrast, primer pair 13–14, designed to target an area 6675 base pairs downstream of primer pair 1–2 and 571 base pairs upstream of exon 1, did not amplify, affirming its position within the deleted region (Figs. 2 and 4). Primer pair 9–10 also provided successful amplification, verifying the integrity of their target region further downstream.

To pinpoint the deletion boundaries more precisely, primer pair 15–16 were introduced, which targeted 1604 base pairs upstream exon 1, beyond the reach of primer pair 13–14. The failure of this pair to amplify reinforced the presence of a deletion within this region (Figs. 2 and 4).

**Fig. 5:**
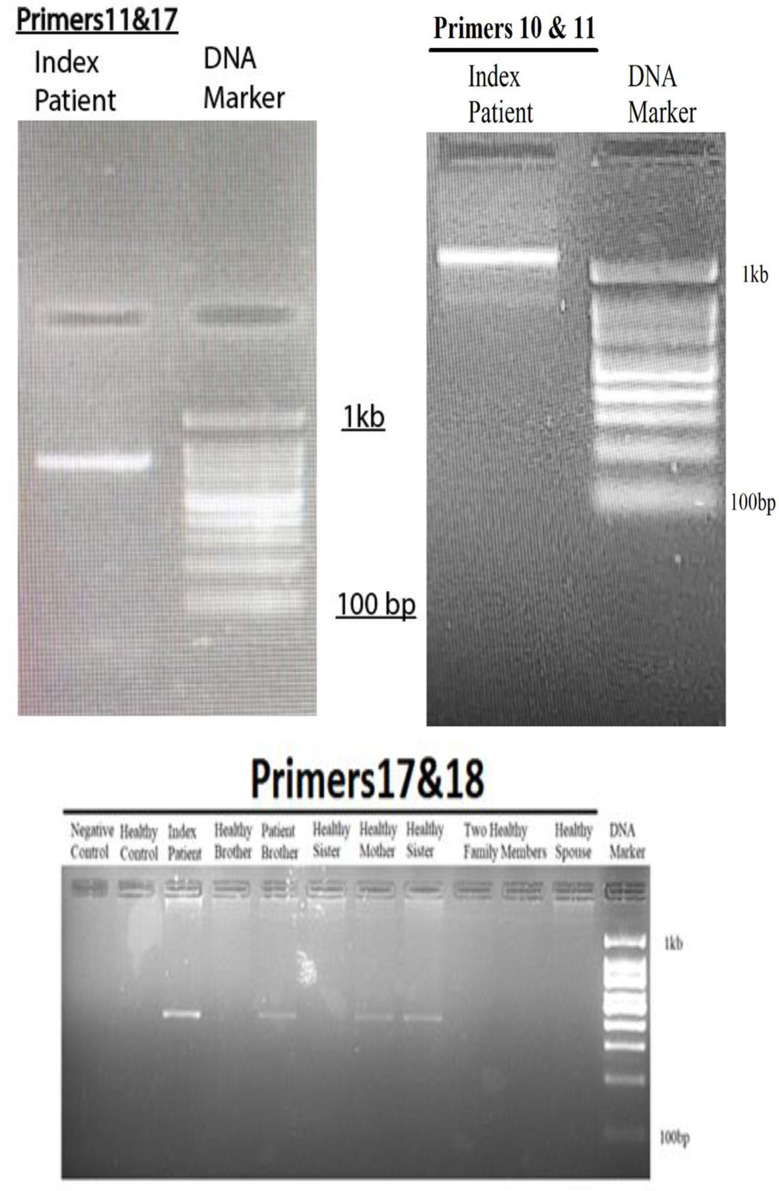
Results of the gel electrophoresis after performing PCR using forward primer from upstream side and reverse primer from downstream side of exon 1 of the *BLOC1S6* gene. To set up the GAP-PCR, we first approached the deletion region as closely as possible using primers 10 and 11. Subsequently, we moved closer to the deletion site, using primers 11 and 17. Finally, we obtained the smallest replicable fragment using primers 17 and 18

Employing Gap-PCR, we used forward primer 11 and reverse primer 10 to initially produce a 1-kilobase PCR product) Fig. 5). Sanger sequencing of this product confirmed its proximity to the deletion boundary, prompting the use of an additional reverse primer 17, which was located 940 base pairs upstream primer 10 (Fig. 2). A subsequent round of GAP-PCR with forward primer 18 and reverse primer 17 yielded an 800-base-pair product, verified through gel electrophoresis, mapping closely to the downstream deletion boundary (Figs. 2 and 4).

The corresponding reference genomic sequence for this region was found to be approximately 7 kb in length when checked with NCBI. It is important to note that a GAP-PCR-based test to detect the deletion relies on the fact that amplification of the wild-type allele is not possible in PCR. Therefore, we can only expect to obtain a PCR product in affected homozygotes and healthy heterozygotes (Figs. 2 and 5).

### Investigation of *BLOC1S6 Gene* expression levels

**Fig. 6:**
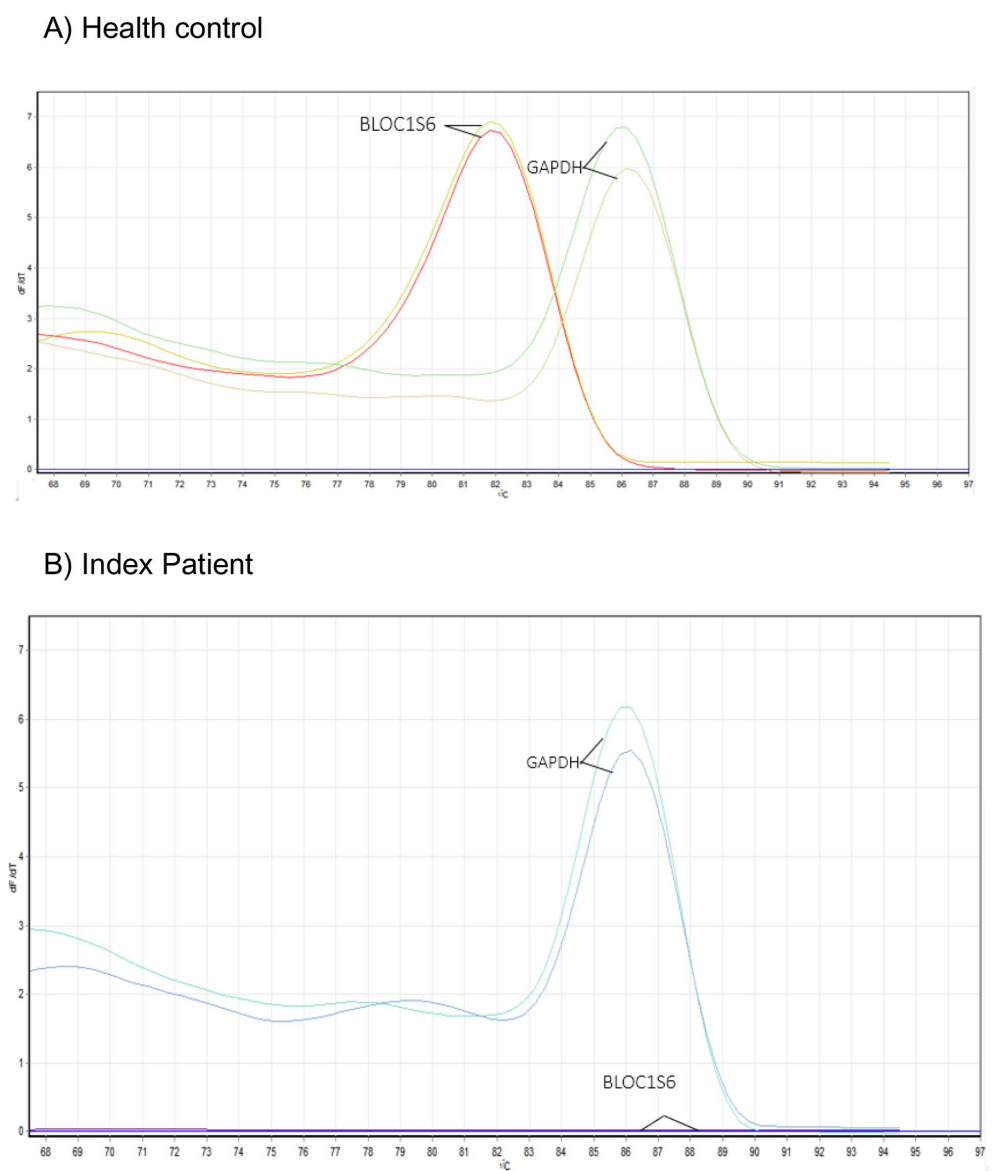
The melt curve analysis for the *BLOC1S6* gene showed no peak, indicated by a horizontal dash at the bottom of the plot. This suggests that there is no alternative transcript of the BLOC1S6 gene that can be expressed, and therefore the *BLOC1S6* gene is not expressed

**Fig. 7:**
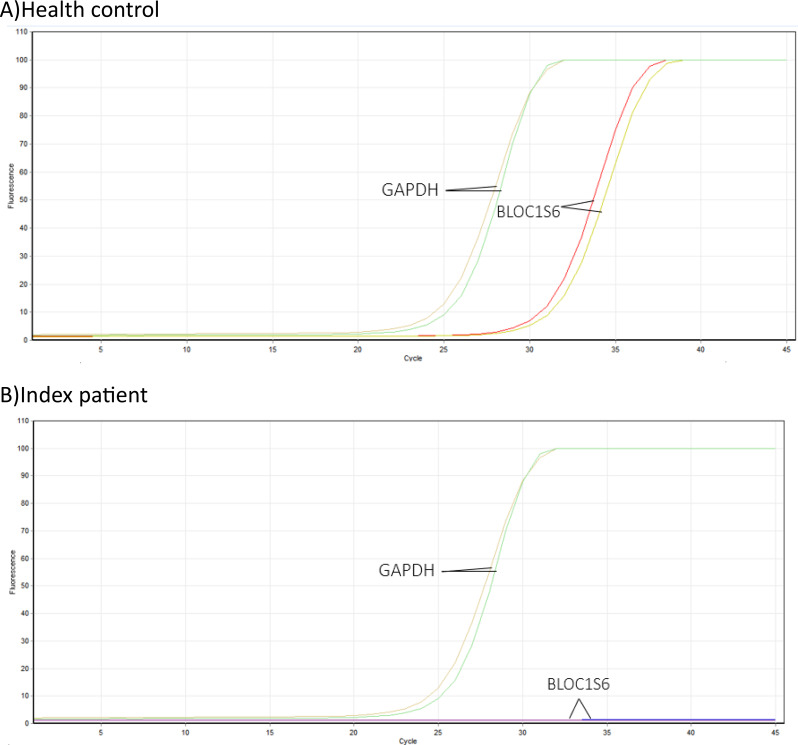
The figure show the examination of the amplification curve for the *BLOC1S6* gene revealed no peak, which is symbolized by a horizontal line at the base of the graph. This absence of a peak suggests that the *BLOC1S6* gene does not have any alternative transcripts that can be expressed. Conversely, the amplification curve analysis for the *GAPDH* gene did show a peak. This is not surprising, as the *GAPDH* gene is a housekeeping gene that is typically found in blood cells. These findings suggest that the *BLOC1S6* gene is not being expressed in the patient’s cells, while the *GAPDH* gene, a housekeeping gene, is being expressed. This could potentially impact the patient’s health condition, given that the patient has a deletion in exon 1 of the *BLOC1S6* gene. The hypothesis was to investigate if there is an alternative transcript that can be expressed despite this deletion. The results indicate that there is no such alternative transcript, which could have significant implications for the patient’s health and treatment

**Fig. 8:**
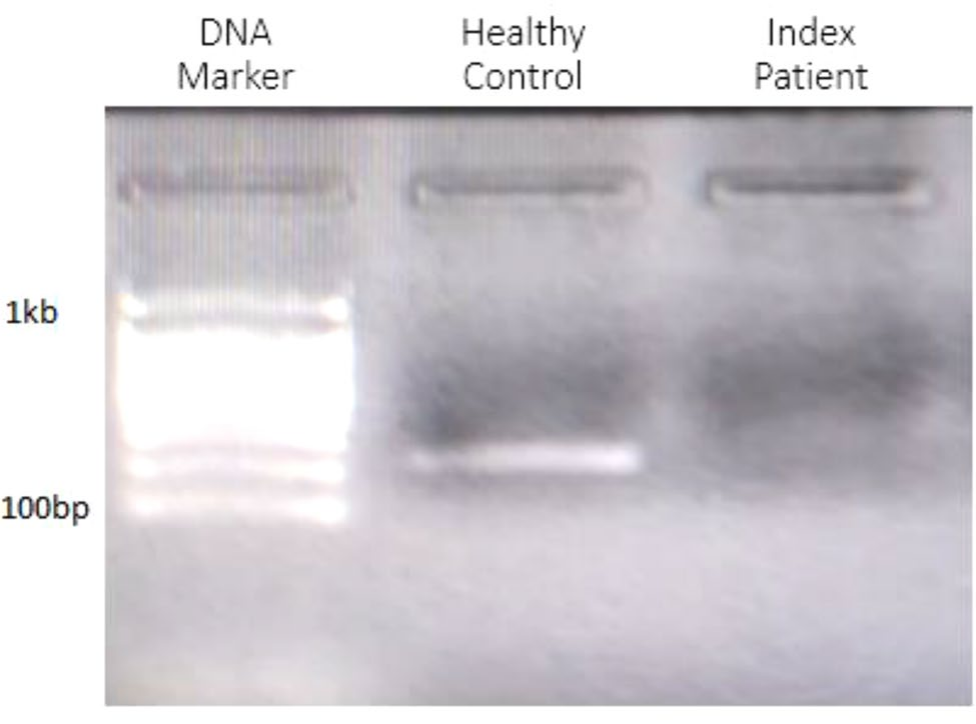
Gel electrophoresis analysis revealing the absence of a product in patient samples compared to healthy control

Real-time PCR experiments were conducted simultaneously in duplicate for both the affected proband and a healthy control. As a result of this investigation, it was determined that the deletion of the first exon of the *BLOC1S6* gene has resulted in the absence of gene expression in the proband. Additionally, the patient did not exhibit any curve in the melt curve and amplification curve during this experiment (Figs. 6 and 7). Furthermore, results of gel electrophoresis analysis revealed the absence of a product in patient samples compared to healthy control (Fig. 8).

These results indicate that the *BLOC1S6* gene is not expressed in the patient’s cells, while the *GAPDH* gene, which is a housekeeping gene, is expressed.

This could have implications for the patient’s health condition, given the deletion of exon 1 in the *BLOC1S6* gene. Our hypothesis was to determine if there is an alternative transcript expressed despite the deletion of exon 1. Based on the findings, no alternative transcripts are seemingly present, which could have significant implications for the patient’s health outcome and treatment.

## Discussion

In the current study, remarkable example of HPS-9 in an Iranian family with two affected siblings was investigated. HPS-9 is a disorder that has only been observed in a small number of cases [23]. HPS-9 belongs to a range of disorders known as Hermansky-Pudlak syndrome. This group of disorders is identified by the presence of abnormalities in the development of organelles related to lysosomes [24]. The underlying mechanism involves variants in genes responsible for the biogenesis of these organelles. In HPS-9, the key genes implicated include *BLOC1S6*, which encodes a subunit of the biogenesis of lysosome-related organelles complex 1 (BLOC-1) [25].

Pallidin and BLOC-1 are essential components of the cellular machinery responsible for the formation and function of lysosome-related organelles [26]. In normal cellular function, these organelles play crucial roles in processes such as melanin synthesis and platelet aggregation [27]. However, in individuals with HPS-9, variants in *BLOC1S6* disrupt the proper functioning of these organelles, leading to the clinical manifestations of the syndrome, including Oculocutaneous Albinism (OCA), bleeding, immunodeficiency, lysosomal ceroid storage, delayed psychomotor development, nystagmus, strabismus, iris trans-illumination, bruising, and a significant reduction in visual acuity [23].

Previous cases of HPS-9 have been reported in diverse geographic regions, including Italy, Pakistan, Japan, Syria, France, and China, revealing a wide spectrum of genetic variants associated with this subtype [12, 23, 28–30] .

**Fig. 9:**
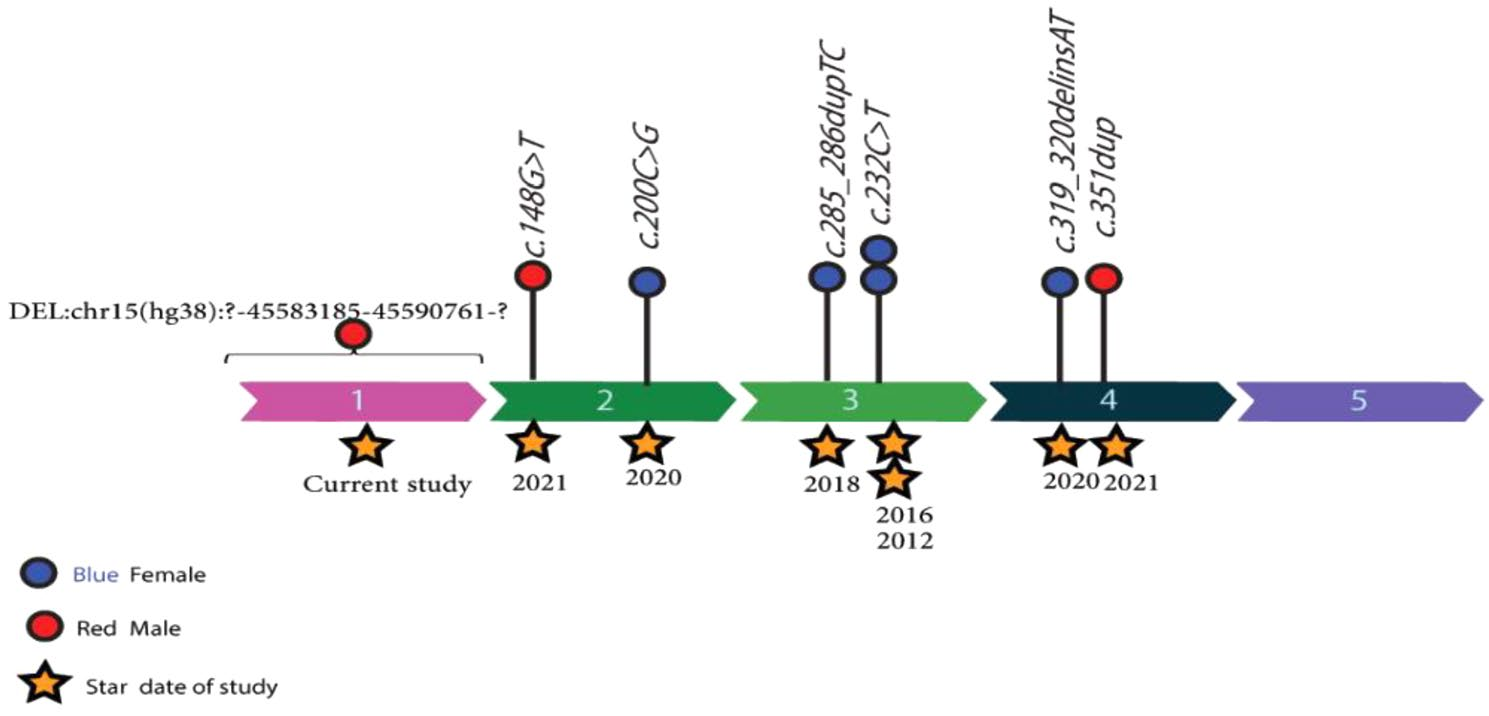
The figure illustrates the positions of identified genetic variants within the exons of the *BLOC1S6* gene. Male cases are represented by red circles, females by blue circles, and the star indicates the date of the study reporting these cases

One such notable case, described by Raffaele Badolato and colleagues in 2012, featured a 17-year-old Italian female with HPS-9. This individual was homozygous for a nonsense variant in exon 3 of the *BLOC1S6* gene (NM_012388.4:c.232C>T, p.Q78*), resulting in the absence of functional BLOC1S6 [12]. The affected individual, similar to our patients, had OCA and impaired visual acuity. However, unlike our patients, this individual did not exhibit any hemorrhagic complications. Moreover, the parents of the affected individual were heterozygous for this particular variant, demonstrating the inheritance pattern typically observed in HPS-9 [12]. In 2016/2017, Sairah Yousaf et al. reported a case of a 4-year-old female with HPS-9 from a Pakistani family that had a known history of HPS-9 [30]. They identified the same variant in the *BLOC1S6* gene that was previously described by Badolato et al. in 2012. This genetic continuity across different populations underscores the pathogenic significance of this specific variant in HPS-9 [12, 30], Also, this case exhibited prolonged bleeding and clotting times, indicating platelet dysfunction in agreement with what we observed in our patients. In 2017/2018, Ken Okamura et al. reported a homozygous variant in the *BLOC1S6* gene (NM_012388.3: c.285_286dupTC, p.H96LfsX22) as the causative genetic alteration for HPS-9 in a 52-year-old Japanese female [29]. This variant led to the dysfunction of pallidin, highlighting the genetic heterogeneity even within the context of HPS-9. This patient was the first among the HPS-9 patients to exhibit symptoms of schizophrenia [29]. Further diversification in genetic alterations was discovered in a 2020 study conducted by Vincent Michaud et al. They reported a 2.5-year-old Syrian female with compound heterozygous variants in *BLOC1S6*, including a nonsense variant in exon 2 (NM_012388.3: c.200C>G, p.Ser67*) and a missense variant in exon 4 (NM_012388.3: c.319_320delinsAT, p.Glu107Met) [28]. The patient exhibits hypopigmentation of the skin and hair, as well as ocular albinism and a bleeding diathesis similar to that of our patients. In contrast to our patients, however, the individual displayed abnormal psychomotor development, experiencing delays in both language acquisition and motor skills development. This case pinpoints the wide spectrum of genetic variants contributing to HPS-9, highlighting the intricate genetic underpinnings of the syndrome [28]. Teng Liu et al. (2021) reported a case of a 6-year-old male from the Chinese population affected by HPS-9. They identified two novel variants in the *BLOC1S6* gene using WES [23]. These variants included a maternal nonsense variant (c.148G > T, p.Glu50*) and a paternal frameshift variant (c.351dupT, p.Ile118Tyrfs*10), both validated by Sanger sequencing. Novel clinical findings in this patient include the detection of abnormal brain waves through electroencephalogram (EEG) and the observation of a tendency towards attention-deficit hyperactivity disorder (ADHD) as indicated by the ADHD diagnostic scale, which was not observed in other patients. This case further accentuates the genetic diversity observed in HPS-9, suggesting that novel variants within the *BLOC1S6* gene continue to be discovered [23]. Interestingly, while visual acuity was a shared characteristic among all known HPS-9 cases, a remarkable observation was made in one of our patients. This patient exhibited a notably enhanced ability to see objects in low-light conditions, especially at night [6, 12, 23, 29, 30].The information pertaining to the current study and the mentioned studies is summarized in Table 2. In addition to the common and similar symptoms summarized in Table 2 for HPS-9 cases, the individuals affected in the pedigree investigated in this study had a tendency to develop skin ulcers. Also the occurrence and placements of discovered genetic variants within the BLOC1S6 exons are shown, with red circles representing males and blue circles indicating females. A timeline marked by stars shows the discovery of these variants from 2012 to 2021. This trend emphasizes the need for more research, especially to comprehend the possible spatial and sex-specific implications of these mutations (See Fig. 9).

**Table 2:** This table presents a comprehensive overview of the phenotypic and genotypic features observed in all reported cases of Hermansky-Pudlak Syndrome 9, up to now, including the findings from the current study

Study by	Badolato et al.	Yousaf et al.	Okamura et al.	Michaud et al.	Liu et al.	Current Study
Publish date	2012	2016	2018	2020	2021	2023
Origin	Italian	Pakistani	Japanese	Syrian	Chinese	Iranian
Gender	Female	Female	Female	Female	Male	Male
Age	17	4	52	2.5	6	32
Hair color	N/A	Golden white	Blond	Yellow	Brownish-yellow	White blonde/ Gold white
Skin color	N/A	Pink white	White	White	White	Pink white
NM	NM_012388.4	NM_012388.4	NM_012388.4	NM_012388.4	NM_012388.4	NM_012388.4
Variant	c.232 C > T	c.232 C > T	c.285_286dupTC	c.200 C > G, c.319_320delinsAT	c.148G > T, c.351dupT	DEL: chr15(hg38):?-45583185-45590761-?
Exon	Exon 3/5	Exon 3/5	Exon 3/5	Exon 2/5, Exon 4/5	Exon 2/5, Exon 4/5	Exon 1/5
Amino acid change	p.Gln78Ter(Q78*)	p.Gln78Ter(Q78*)	p.His96LeufsTer22	p.Ser67Ter(S67*), p.Glu107Met (E107M)	p.Glu50Ter(E50*), p.Ile118TyrfsTer10(I118Yfs*10)	NMD or Unknown
Protein Position	78/173	78/173	96 /173	67/173, 107/173	50/173, 118/173	1–28/173
Frequency (ExAC)	< 0.01%	< 0.01%	N/A	< 0.01%, N/A	N/A, N/A	N/A
Zygosity	Homo	Homo	Homo	Compound heterozygous	Compound heterozygous	Homo
Variant type	Nonsense	Nonsense	Frameshift	Nonsense, Missense	Nonsense, Frameshift	CNV (deletion)
ACMG classification	Pathogenic	Pathogenic	Likely Pathogenic	Pathogenic, Likely Pathogenic	Pathogenic, Pathogenic	Likely Pathogenic
CLINVAR	Pathogenic	Pathogenic	Not registered in Clinvar	Pathogenic, Likely Pathogenic	Not registered in Clinvar, Not registered in Clinvar	Not registered in Clinvar
Inheritance	AR	AR	AR	AR	AR	AR

Confirming the large deletion identified in our case presented significant challenges in terms of experimental methods. Traditional Sanger sequencing alone is insufficient because it requires knowledge of approximate deletion boundaries before starting the sequencing process [31]. Therefore, we employed a combination of primer walking and GAP-PCR methods to approximate and confirm the deletion boundaries [32]. our approach was initiated with the primer walking method, advancing as far as possible to amplify both sides of the deletion region and generate a PCR product. The GAP-PCR method was then employed, strategically placing a pair of primers on either side of the deletion. This method resulted in an approximately 500-base-pair PCR product, whereas in a healthy individual, the expected genomic span of this region is around 7 kilobases. Through these techniques, it became evident that approximately 6,500 base pairs were homozygously deleted in the proband.

This innovative approach underscores the necessity for specialized techniques to confirm and validate CNVs within the genetic landscape of HPS-9 and similar disorders. Moreover, it highlights the significance of conducting in-depth genetic analyses to fully comprehend the underlying genetic variants contributing to rare and complex diseases such as HPS-9.

In conclusion, our study not only contributes to the expansion of knowledge regarding HPS-9 etiology but also highlights the genetic heterogeneity of this syndrome. The discovery of a novel CNV in an Iranian family with HPS-9 showcases the complexity of genetic alterations associated with this disorder and highlights the need for further research to elucidate the complete spectrum of variants that contribute to HPS-9. As our knowledge of the genetic basis of HPS-9 continues to expand, we may move closer to improved diagnostic and therapeutic approaches for individuals affected by this rare and challenging syndrome.

## Data Availability

The data that support the findings of this study are available from the corresponding author upon reasonable request.
